# Lower access to risk stratification tests and drugs, and worse survival of chronic lymphocytic leukaemia patients treated in public as compared to private hospitals in Brazil: A retrospective analysis of the Brazilian registry of chronic lymphocytic leukaemia

**DOI:** 10.1002/jha2.444

**Published:** 2022-05-06

**Authors:** Verena Pfister, Fernanda de Morais Marques, Flavia Parra, Mihoko Yamamoto, Matheus Vescovi Gonçalves, Leila Perobelli, Valeria Buccheri, Raphael Bandeira, Sergio Fortier, Alita Azevedo, Rodrigo Santucci, Marcelo Bellesso, Laura Fogliatto, Glaciano Ribeiro, Germison Silva Lopes, Maura Ikoma, Vera P. Figueiredo, Irene Gyongyver H Lorand Metze, Carlos Sérgio Chiattone, Celso Arrais‐Rodrigues

**Affiliations:** ^1^ Escola Paulista de Medicina /Universidade Federal de São Paulo, UNIFESP São Paulo Brazil; ^2^ Brazilian Registry of CLL – Associação Brasileira de Hematologia e Hemoterapia Hemoterapia Brazil; ^3^ Hospital Brigadeiro São Paulo Brazil; ^4^ ICESP Faculdade de Medicina da USP São Paulo Brazil; ^5^ Santa Casa de São Paulo São Paulo Brazil; ^6^ HEMOPE Recife Brazil; ^7^ Hemomed São Paulo Brazil; ^8^ Hospital das Clínicas de Porto Alegre Porto Alegre Brazil; ^9^ Universidade Federal de Minas Gerais Belo Horizonte Brazil; ^10^ Universidade Federal do Ceará Fortaleza Brazil; ^11^ Hospital Amaral Carvalho Jaú Brazil; ^12^ Hospital do Servidor Público Estadual São Paulo Brazil; ^13^ Universidade Estadual de Campinas, UNICAMP Campinas Brazil; ^14^ Hospital Samaritano São Paulo Brazil; ^15^ Hospital 9 de Julho São Paulo Brazil

**Keywords:** CLL, public health system, risk stratification

## Abstract

Chronic lymphocytic leukaemia (CLL) has a highly variable clinical course. In addition to biological factors, socioeconomic factors and health system characteristics may influence CLL outcome. Data from the Brazilian Registry of CLL were analyzed to compare clinical and treatment‐related characteristics in patients with CLL, from public or private institutions. A total of 3326 patients from 43 centres met the eligibility criteria, of whom 81% were followed up at public hospitals and 19% at private hospitals. The majority were male (57%), with a median age of 65 years. Comparing public and private hospitals, patients in public hospitals were older, had more advanced disease at diagnosis, and more frequently had elevated creatinine levels. All investigated prognostic markers were evaluated more often in private hospitals. First‐line treatment was predominantly based on chlorambucil in 41% of the cases and fludarabine in 38%. Anti‐CD20 monoclonal antibody was used in only 36% of cases. In public hospitals, significantly fewer patients received fludarabine‐based regimens and anti‐CD20 monoclonal antibodies. Patients from public hospitals had significantly worse overall survival (71% vs. 90% for private hospitals, *p* < 0.0001) and treatment‐free survival (32% vs. 40%, for private hospitals, *p* < 0.0001) at seven years. Our data indicate striking differences between patients followed in public and private hospitals in Brazil. A worse clinical condition and lack of accessibility to basic laboratory tests and adequate therapies may explain the worse outcomes of patients treated in public institutions.

## 1 INTRODUCTION

Chronic lymphocytic leukaemia (CLL) is the most common type of leukaemia in adults in Western countries. Considerable advances have been made in understanding the biology of CLL and the use of prognostic markers to predict disease progression and therapeutic outcomes. Interpretation of international guidelines still varies considerably, specifically regarding when to initiate CLL therapy, how to apply prognostic factors when making treatment choices, and the type and sequence of therapeutic regimens offered to patients [1, 2, 3, 4, 5]. Immunoglobulin heavy chain locus (IGHV) mutational status and abnormalities detected by fluorescence in situ hybridisation (FISH) have been shown to predict survival in patients with CLL [6]. They are not recommended to drive treatment initiation decisions [1] but can guide follow‐up intervals for high‐risk patients [6]. CLL is a disease that predominantly affects elderly people, and the management of elderly patients with CLL is more complex than that of younger patients because of a greater frequency of comorbidities [7, 8]. Furthermore, differences in patient outcomes can exist between those treated in clinical trials and those treated in clinical practice: patients in clinical trials are usually younger, have fewer comorbidities, have more favourable Eastern Cooperative Oncology Group Performance Status, and different racial and/or socioeconomic profiles [9]. Besides, the type of area (rural or urban) and type of hospital may influence response and survival in CLL [10, 11].

Therefore, the treatments offered to patients with CLL and the resulting outcomes may vary considerably among institutions, as well as among academic, community, private, or public settings.

Here, we describe the clinical characteristics, prognostic markers, and treatment patterns of patients followed in public or in private centres in Brazil.

## 2 METHODS

### 2.1 Study design and participants

The Brazilian Registry of CLL was started in 2004 as a prospective non‐interventional data collection tool to gather information about the real‐life experiences of patients with CLL. The Brazilian Registry of CLL is a multicentre, ambispective, observational cohort study. Sites were encouraged to enrol all patients consecutively, including newly diagnosed and patients that were diagnosed in the past and presented for their follow‐up visits. Registered patients are required to have a diagnosis of CLL, as defined by the International Workshop on CLL (IWCLL) guidelines [12], confirmed by multiparametric flow cytometry. Only patients who had a date of birth, date of diagnosis, date of the last follow‐up, or date of death were eligible for inclusion. The minimum follow‐up time required for inclusion was three months.

### 2.2 Assessments

Information was collected via an electronic data capture system and included demographic information, relevant medical history, laboratory testing, available diagnostic flow cytometry analyses, and prognostic testing (FISH, *IGHV* mutational status, karyotype and beta‐2 microglobulin).

To analyse the frequency of different treatment strategies, we analysed 1255 patients who were treated between January 2008 and October 2021. We chose this interval because there were significantly less missing data in patients treated after 2007 than those treated in the preceding years.

### 2.3 Statistical analysis

Statistical analyses to assess differences in characteristics at enrolment between patient subgroups (private or public) were conducted using a chi‐square test, for the comparison of rates, and a *t*‐test for the comparison of medians. The median and range were used for the descriptive analysis of continuous variables.

The probabilities of overall survival (OS) and treatment‐free survival (TFS) were calculated using the Kaplan‐Meier estimator and compared using the log‐rank test. Univariate and multivariate Cox regression analyses were performed to determine the independent risk indicators for OS and TFS. OS was defined as the interval between the date of diagnosis and the date of the last follow‐up or death. TFS was defined as the interval between the date of diagnosis and the date of start of first‐line treatment, date of the last follow‐up, or death.

All statistical significance was assessed at a 5% level (two‐sided). Statistical analyses were performed using SPSS 20 (SPSS, Chicago, IL) and R 2.13.2 (R Development Core Team, Vienna, Austria) software packages.

## 3 RESULTS

### 3.1 Patient population

Between January 2004 and October 2021, 3476 patients were enrolled in the Brazilian Registry of CLL from 43 centres throughout Brazil (30 public and 13 private). A total of 3326 patients (96%) fulfilled the minimum required data for analysis and were eligible for inclusion.

Table 1 provides the demographics and characteristics of the total analysed population and of patients from public or private hospitals. Of the 3326 included, 2695 patients (81%) were from public centres and 631 (19%) were from private centres. The median age was 65 years (range 23–106 years), and most patients were male (57%). Binet stage at diagnosis was A in 1844 (59%) patients, B in 715 (23%), and C in 573 (18%).

**TABLE 1:** Patient demographics and characteristics at diagnosis

Characteristics at diagnosis	All patients	Public	Private	*p*
Patients – *n* (%)	3326	2695 (81%)	631 (19%)	
Male sex – *n* (%)	1880 (57%)	1510 (56%)	370 (59%)	0.19
Age, years – median (range)	65 (23–106)	66 (23–106)	63 (31–98)	<0.0001
Binet staging				<0.0001
A – *n* (%)	1844 (59%)	1450 (57%)	394 (67%)	
B – *n* (%)	715 (23%)	576 (23%)	139 (24%)	
C – *n* (%)	573 (18%)	521 (20%)	52 (9%)	
Haemoglobin, g/dl – median (range)	13.0 (2.5–19.0)	13.0 (2.5–19.0)	13.7 (3.9–18.0)	<0.0001
Lymphocytes, /mm^3^‐ median (range)	22,100 (5027–953800)	25,382 (5027–953,800)	12,111 (5030–363,000)	<0.0001
Platelets, /mm^3^‐ median (range)	180,000 (1400–689000)	176,000 (1400–689,000)	195,000 (3540–619,000)	<0.0001

Comparing public and private hospitals, we observed that patients in public hospitals were significantly older (median age 66 years vs. 63 years for private hospitals, *p* < 0.0001), had more advanced disease at diagnosis (frequency of Binet B or C was 44% in public vs. 33% in private hospitals, *p* < 0.0001), and more frequently had creatinine levels above the reference values (18% vs. 10%, *p* < 0.0001).

We then analysed the frequency of prognostic factors that were evaluated at any time before the first‐line treatment (Table 2). FISH for del(17p) was performed in only 559 patients (17%), whereas FISH for the most common aberrations [del(13q), +12, del(11q), and del(17p)] was performed in only 471 patients (14%). IGHV mutational status was evaluated in only 285 patients (8.5%), karyotype in only 491 patients (15%) and beta‐2 microglobulin in 1168 (35%).

**TABLE 2:** Frequency of prognostic factor tests before first‐line treatment

Prognostic factors	All patients (*n* = 3326)	Public (*n* = 2695)	Private (*n* = 631)	*p*
FISH for 17p – *n* (%)	559 (17%)	275 (10%)	284 (45%)	<0.0001
FISH for 17p only – *n* (%)	88 (3%)	53 (2%)	35 (5%)	
FISH for CLL panel* – *n* (%)	471 (14%)	222 (8%)	249 (40%)	<0.0001
*IgHV* – *n* (%)	285 (8.5%)	167 (6%)	118 (19%)	<0.0001
Beta‐2 microglobulin	1168 (35%)	869 (32%)	299 (47%)	<0.0001
Karyotype – *n* (%)	491 (15%)	337 (12.5%)	154 (24%)	<0.0001
Molecular tests – *n* (%)	44 (1%)	15 (0.5%)	29 (5%)	<0.0001

* Including 13q deletion, 12 trisomy, 11q deletion, and 17p deletion.

All investigated prognostic markers were more often evaluated in private hospitals than in public hospitals: FISH for del(17p) (45% vs. 10%, respectively, *p* < 0.0001), *IGHV* mutational status (19% vs. 6%, respectively, *p* < 0.0001), karyotype (24% vs. 12.5%, respectively, *p* < 0.0001), and beta‐2 microglobulin (47% vs. 32%, *p* < 0.0001, Table 2). Besides, patients who had available FISH results were younger (62 vs. 66 years, *p* = 0.03), and had the more advanced disease by Binet (19% for Binet B or C vs. 16% for Binet A, *p* < 0.0001).

The FISH test positivity rate for del(17p) was similar between public and private hospitals (10.5% vs. 9%, *p* = 0.67), as was the frequency of unmutated *IGHV* status (50% vs. 56%, *p* = 0.26). However, elevated beta‐2 microglobulin was slightly more frequent in patients from public centres (46% vs. 40% in private centres, *p* = 0.08, Table 3).

**TABLE 3:** Frequency of adverse prognostic factors before first‐line treatment

Prognostic factor	All patients	Public	Private	*p*
FISH del(17p) – *n* (%)	55 (10%)	29 (10.5%)	26 (9%)	0.67
*IgHV* Unmutated – *n* (%)	149 (52%)	83 (50%)	66 (56%)	0.26
Elevated beta‐2 microglobulin – *n* (%)	517 (44%)	398 (46%)	119 (40%)	0.08

CLL‐IPI (international prognostic index for chronic lymphocytic leukaemia) was calculated for all patients with available information. Unfortunately, all five risk factors (age, Binet, beta‐2 microglobulin, *IGHV* mutational status and del(17p)/*TP53*) were identified in only 130 patients (4%). However, in 432 patients (13%), we were able to stratify patients according to CLL‐IPI with only one of the following risk factors: 175 (40%) had a low or intermediate score and 257 (60%) had a high or very high score (Table 4). There were significantly more patients with high or very high CLL‐IPI scores in public centres (69% vs. 45% in private centres, *p* < 0.0001).

**TABLE 4:** Chronic lymphocytic leukaemia (CLL)‐IPI risk groups

	All patients	Public	Private	*p*
CLL‐IPI risk score	432	263	169	
Low risk (0 or 1)	83 (19%)	35(13%)	48 (28%)	<0.0001
Intermediate risk (2 or 3)	92 (21%)	47 (18%)	45 (27%)	
High risk (4 to 6)	244 (57%)	174 (66%)	70 (41%)	
Very high risk (7 to 10)	13 (3%)	7 (3%)	6 (4%)	
CLL‐IPI groups	432	263	169	
Low or intermediate risk	175 (40%)	82 (31%)	93 (55%)	<0.0001
High or very high risk	257 (60%)	181 (69%)	76 (45%)	

**FIGURE 1:**
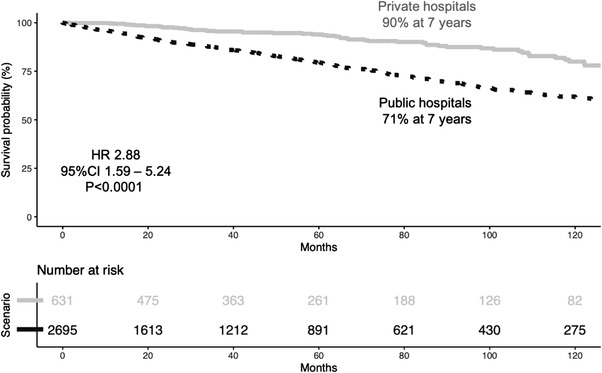
Overall survival of patients with CLL in private (^___^) and public (‐ ‐ ‐) hospitals

The median follow‐up time was 47 months (range: 3‐316 months), and the 75^th^ percentile follow‐up time was 88 months (7 years and 4 months). The median OS was not reached, and the OS rate was 75% at seven years. OS was significantly worse in public than in private hospitals (71% vs. 90%, respectively, *p* < 0.0001, Figure 1).

In a multivariate analysis, OS in patients from public hospitals remained significantly worse than in private hospitals (hazard ratio – HR 2.36, 95% confidence interval – 95%CI 1.49–3.74), after correcting for age (older than 65 years), Binet staging (B or C vs. A), and elevated creatinine.

**FIGURE 2:**
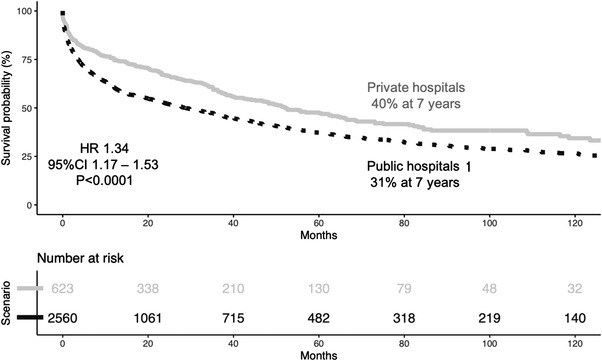
Treatment‐free survival of patients with CLL in private (^___^) and public (‐ ‐ ‐) hospitals

The TFS was 33% at seven years. The median TFS was 35 months, and TFS was significantly worse in public than in private hospitals (32% vs. 40%, respectively, *p* < 0.0001, figure 2). The median TFS was 29 months for public centres and 52 months for private centres. In a multivariate analysis, TFS in patients from public hospitals remained significantly worse than in private hospitals (HR 1.34, 95%CI 1.17–1.53), after correcting for age (older than 65 years), and Binet staging (B or C vs. A).

After analysing 1255 patients who had been treated after January 2008, treatment was initiated after a median time of seven months (range: 0‐290) after diagnosis. Among the 1255 treated patients, FISH for del(17p) was performed before treatment in only 285 patients (23%), while IGHV mutational status was performed in only 240 patients (19%) and beta‐2 microglobulin in 478 (38%). As observed for the whole population, among treated patients, most prognostic markers were more often performed in private than in public hospitals: FISH for del(17p) (58% vs. 14%, respectively, *p* < 0.0001) and IGHV mutational status (31% vs. 16%, respectively, *p* < 0.0001). The frequency of beta‐2 microglobulin determination was similar between private and public hospitals (44% vs. 47%, respectively).

First‐line treatment was predominantly based on chlorambucil or fludarabine, which were prescribed to a total of 1000 patients (79%) (Table 5): chlorambucil in 41% of patients and fludarabine in 38%. An anti‐CD20 monoclonal antibody was used in only 36% of cases (rituximab, 32%; obinutuzumab, 4%). Novel agents were used in the first line in only 64 patients (5%), 19 of which were used in the context of a clinical trial. Only 5% of patients were treated in the context of interventional clinical trials.

**TABLE 5:** Time‐to‐treatment and treatment approaches in 1080 patients treated after January 2008

Therapy	All patients	Public	Private	*p*
Treated patients – *n* (%)	1255	1004	251	
Time to treatment, months – median (range)	7 (0–290)	6 (0–207)	13 (0–290)	0.001
Chlorambucil‐based	518 (41%)	444 (44%)	74 (29%)	<0.0001
Fludarabine‐based	482 (38%)	362 (36%)	120 (48%)	<0.0001
CHOP/CVP‐based	152 (12%)	139 (14%)	13 (5%)	<0.0001
Other regimens:				
Bendamustine‐based	28 (2%)	4 (0.4%)	14 (6%)	<0.0001
Venetoclax	28 (2%)	15 (1%)	13 (5%)	0.0001
Acalabrutinib*	23 (2%)	15 (1%)	8 (3%)	0.07
Ibrutinib	15 (1%)	4 (0.4%)	11 (4%)	<0.0001
Others**	7 (0.5%)			
Use of anti‐CD20 antibodies	453 (36%)	264 (26%)	189 (75%)	<0.0001
Rituximab	404 (32%)	240 (24%)	164 (65%)	<0.0001
Obinutuzumab***	46 (4%)	21 (2%)	25 (10%)	<0.0001
Ofatumumab*	3	3	0	<0.0001

* All in the context of the clinical trial.

** Others: cyclophosphamide, rituximab, lenalidomide, steroids, splenectomy.

*** 20/46 in the context of clinical trials.

As expected, fludarabine‐based therapies were more often used in patients aged < 65 years (58% of cases) than in those over 65 years (16%), while the opposite was observed with chlorambucil‐based regimens (21% in patients < 65 years versus 65% in older patients).

In public hospitals, there were significantly fewer patients who received fludarabine‐based regimens as compared to private hospitals (36% vs. 48%, respectively, *p* < 0.0001), and significantly fewer patients received anti‐CD20 monoclonal antibodies (26% vs. 75%, respectively, *p* < 0.0001). Nevertheless, there were relatively more patients over the age of 65 who received fludarabine in public hospitals (32%) than in private hospitals (14%). Surprisingly, most patients with del(17p) or TP53 mutations (69%) received chemoimmunotherapy as first‐line therapy.

Among the patients treated since January 2008, the median follow‐up time after treatment was 39 months (range: 3–160 months). OS after treatment initiation was 71% at five years, and survival after treatment was also significantly worse in public than in private hospitals (68% vs. 82%, respectively, *p* = 0.002).

## 4 DISCUSSION

In this retrospective study of the Brazilian Registry of CLL, we analysed the clinical and laboratory characteristics of a large group of patients with CLL and observed striking differences between patients treated in public and private centres in Brazil. Namely, FISH for del(17p), IGHV mutational status and karyotype were performed in a small percentage of patients before first‐line treatment, especially in the public setting. In public hospitals, there were significantly fewer patients who received fludarabine‐based regimens and regimens containing anti‐CD20 monoclonal antibodies. The lack of prognostic markers and reduced access to appropriate treatment probably reflected the worse survival of the group of patients from public centres.

The Brazilian Registry of CLL made a huge effort to enrol patients who were representative of the Brazilian CLL population and include patients from multiple geographically diverse regions. Patients were enrolled from a large number of sites distributed across the country. However, the sites were still concentrated in the Southeast region, where most reference centres are located. Further efforts are needed to increase the inclusion of patients from less‐resourced regions, such as the North and Northeast regions. Besides, the type of area (rural or urban) and type of hospital may influence response and survival in CLL [10, 11]. An analysis from the Swedish national registries revealed significantly worse OS and PFS in rural/county hospitals than in university/regional hospitals [11].

To minimise bias and better understand the patient population included in our registry, sites were instructed to preferably enrol patients as they are diagnosed, while also including every patient at the time of their medical visit, regardless of disease status or treatment line.

Overall, the Brazilian Registry of CLL appears to be representative of the Brazilian CLL population. As previously observed in a preliminary analysis of the registry [13], the median age of patients enrolled in the Brazilian Registry of CLL was 65 years, whereas the median age of patients at diagnosis was reported in the Surveillance, Epidemiology, and End Results (SEER) Program was 71 years ( https://seer.cancer.gov/csr/1975_2007 ), which could be explained by a significant gap between life expectancy in Brazil (75.9) and the United States (78.5) (World Health Statistics: Monitoring health for the SDGs. https://www.who.int/data/gho/data/indicators/indicator‐details/GHO/ ) [14]. As CLL is a disease of the elderly, a shorter life expectancy might be associated with a lower incidence of CLL because fewer people live long enough to be exposed to the risk of developing the disease. Another possible reason for the younger age might be the ethnic composition of our population, largely represented by mestizos, African‐American and Native‐American descendants. At last, this could also be due to a referral bias leading to underdiagnosis of CLL, probably much more common in the public setting. The majority of patients in the Brazilian Registry of CLL were followed up at public centres. Indeed, only 22.3% of the Brazilian population has private insurance, according to national governmental data (SIB/ANS/MS – Sistema de Informações de Beneficiários‐SIB/ANS/MS. https://www.ans.gov.br/perfil‐do‐setor/dados‐gerais). Our public health system is unfortunately still quite inefficient for diagnosing and treating oncology patients in general, and many patients die in rural and poorer parts of the country without access to diagnosis. In addition, the vast majority of patients (95%) were being cared for outside the context of interventional clinical trials, thereby allowing insight into the management of CLL. As such, the registry provides detailed, patient‐level, real‐world observational data on a diverse population of patients with CLL in Brazil.

Genomic aberrations in CLL are important predictors of disease progression and survival, and unfavourable prognostic markers can be easily identified using karyotyping or FISH [15, 16, 17]. The National Comprehensive Cancer Network ([18, 19]; https://www.nccn.org/), the IWCLL [12] guidelines, and a recent meta‐analysis of genetic testing in newly diagnosed CLL [6] recommend prognostic testing, such as FISH and IGHV, in the management of patients with CLL. However, limited information exists regarding how these guidelines can be interpreted in practice. In the Brazilian Registry of CLL, genetic testing by FISH and karyotyping was performed in only 17% and 15% of patients, respectively, and only 8.5% of patients were tested for IGHV somatic hypermutation, with significantly less testing in public than in private hospitals. In the Swedish Registry Analysis, after FISH was recommended in the national guidelines, there has been a significant increase in the frequency of cytogenetic analysis [11]. Besides, cytogenetic analysis has been more often performed at university hospitals than in other types of hospitals [11].

These data suggest a very low frequency of essential genetic testing in Brazil, possibly due to the unavailability of tests in most centres, but might also reflect the need for more medical education initiatives to promote the learning of adequate risk stratification and therapy adjustments in CLL. The Brazilian Group of CLL is promoting several initiatives to help guide clinicians for a more accurate diagnosis, risk stratification and treatment of CLL patients, such as the Brazilian Guidelines for CLL [20], currently being updated to be published in the next months.

In Brazil, although kinase inhibitors are currently available only in private centres, the correct risk stratification using genetic testing dramatically affects treatment selection in all lines of therapy. For example, in our registry, most patients with 17p deletion and/or *TP53* mutations were not identified and were probably treated with chemoimmunotherapy, although it is widely known to be ineffective in that context [2, 20, 21]. In the present analysis, we were surprised to observe that nearly 70% of patients with del(17p), were treated with chemoimmunotherapy. This was probably due to delayed availability of the result (very common in public centres) but also due to the unavailability of novel agents in both scenarios, forcing the clinicians to use the only available treatment in their centres: chemotherapy or chemoimmunotherapy. Indeed, the Brazilian national regulatory agency (ANVISA) approved ibrutinib in 2015 and venetoclax in 2018, but only after February 2021 insurances companies were obliged to cover these drugs. In the public, neither ibrutinib nor venetoclax is approved to date yet, unfortunately, for any line of treatment.

Moreover, even with targeted agents, patients with high‐risk cytogenetics by FISH or karyotype have been shown to have worse PFS than their low‐risk counterparts [22, 23, 24, 25, 26].

Although chemoimmunotherapy combinations, particularly fludarabine, cyclophosphamide and rituximab (FCR) have been considered the standard of care for many years in the front‐line treatment of fit patients with CLL, only 58% of patients < 65 years of age in the Brazilian Registry of CLL were treated with purine analogue‐based regimens, while the remainder was mostly treated with a combination of chlorambucil and rituximab or with cyclophosphamide, doxorubicin, vincristine and prednisone (CHOP)/cyclophosphamide, vincristine and prednisone (CVP)‐based regimens. On the other hand, there were more patients over the age of 65 who received fludarabine in public hospitals (32%) than in private hospitals (14%). This finding is probably explained by the fact that, as not even rituximab or obinutuzumab is available in most public centres, some elderly patients might be receiving FC (fludarabine and cyclophosphamide) in the public, and not chlorambucil monotherapy, in an attempt to offer a more effective regimen in first‐line, as second‐line options available (basically the same agents – fludarabine or chlorambucil) are also very ineffective. Therefore, clinicians in public centres might have been trying to extend the maximum age that they believe would benefit from a fludarabine‐based regimen.

As most patients were treated in public centres and enrolment in clinical trials was very low, very few patients (3%) were treated with kinase inhibitor therapies as first‐line treatment.

The striking differences observed between patients and outcomes from public and private hospitals are probably due to important factors that determine patterns of routine clinical care, especially in less‐resourced countries. Such factors include striking socioeconomic differences between patients treated in both scenarios, regulatory actions, infrastructure development, and, most importantly, striking differences in funding. In addition, the current analysis raises the question as to whether results obtained from clinical trials can be extrapolated to clinical practice in Brazil, in which correct prognostication and adequate treatment are unattainable for more than 75% of the population.

While analysing the data, we observed several data quality issues that may affect the validity of our findings, which include limited data availability, as most centres lack adequate data management, and underreporting of outcomes, as many patients lose follow‐up. These are clearly critical areas for further enquiry and deserving of improvement. Furthermore, potential sources of bias are inherent to any registry‐based study arising from data collection and assessment of treatment effectiveness due to lack of randomisation.

In conclusion, in this analysis of the Brazilian registry of CLL data, important trends in real‐world practice patterns, including infrequent use of prognostic testing, marked underutilisation of novel agents and a very low rate of participation in first‐line interventional clinical trials were observed. We observed striking differences between patients treated in public or private hospitals in Brazil. A worse clinical condition as well as the lack of accessibility to basic laboratory tests and adequate therapies may explain the worse outcome of patients treated in public institutions.

These findings may provide physicians from less‐resourced countries with a greater understanding of the realities of treating patients with CLL and the challenges commonly encountered in everyday practice. These outcomes will provide further insights and information to those involved in the community‐based care of patients with CLL.

## CONFLICT OF INTEREST

The authors declare they have no conflicts of interest.

## AUTHOR CONTRIBUTIONS

**Conception and design**: Verena Pfister, Mihoko Yamamoto, Matheus Vescovi Gonçalves, Carlos Sérgio Chiattone and Celso Arrais‐Rodrigues. **Collection and assembly of data**: Verena Pfister, Fernanda de Morais Marques, Flavia Parra, Leila Perobelli, Valeria Buccheri, Raphael Bandeira, Sergio Fortier, Alita Azevedo, Rodrigo Santucci, Marcelo Bellesso, Laura Fogliato, Glaciano Ribeiro, Germison Silva Lopes, Maura Ikoma, Vera P. Figueiredo, Irene Gyongyver H Lorand Metze, Carlos Sérgio Chiattone and Celso Arrais‐Rodrigues. **Data analysis and interpretation**: Verena Pfister, Mihoko Yamamoto, Matheus Vescovi Gonçalves, Carlos Sérgio Chiattone and Celso Arrais‐Rodrigues. **Manuscript writing**: All authors. **Final approval of manuscript**: All authors. **Accountable for all aspects of the work**: All authors.
